# Editorial: Advances in additive manufacturing technologies for the production of tissue-engineered bone scaffolds for dental applications

**DOI:** 10.3389/fbioe.2022.980430

**Published:** 2022-08-09

**Authors:** Giulia Brunello, Nikolaos Donos, Stefano Sivolella, Barbara Zavan

**Affiliations:** ^1^ Department of Oral Surgery, University Hospital of Düsseldorf, Düsseldorf, Germany; ^2^ Department of Neurosciences, University of Padua, Padua, Italy; ^3^ Centre for Oral Clinical Research, Barts and the London School of Medicine and Dentistry, Institute of Dentistry, Queen Mary University of London, London, United Kingdom; ^4^ Department of Medical Sciences, University of Ferrara, Ferrara, Italy

**Keywords:** bone regeneration, 3D printing, biocompatibility, regenerative medicine, personalized therapy, additive manufacturing

In the attempt to repair bone tissue defects, a plethora of bone substitute materials of various origins have been utilized so far. Despite the fact that autogenous, allogenic and xenogeneic bone grafts still constitute valid therapeutic options in daily practice, bone tissue engineering strategies have gained popularity for the regeneration of damaged tissues (Cao et al.). Cells, scaffolds and growth factors represent the key elements of tissue engineering. Due to the ability of the bone to self-regenerate due to the migration of cells and growth factors from the adjacent tissues, the success of bone tissue engineering largely relies in the ability of the scaffold to act as a template for guiding tissue regeneration. Indeed, not only cell-laden scaffolds, but also cell-free biomimetic matrixes are considered appealing solutions in bone regeneration, as they can stimulate cell colonization and recruitment from neighbouring tissues. In this context, additive manufacturing (AM) technologies have gained considerable interest owing to their versatility and the possibility to produce personalized scaffolds with complex geometry, matching the patient’s bone defects (Brunello et al.; Latimer et al.).

Although several endeavours intended to promote vertical bone augmentation have been reported, they are characterized by inconsistent long-term results and varying degrees of success (Retzepi and Donos, 2010; Donos et al., 2015; Urban et al., 2019). AM is endorsing novel alveolar ridge augmentation strategies for vertical bone gain, aiming at achieving prolonged volumetric space maintenance during extra-skeletal bone remodelling and maturation (Vaquette et al.). Nevertheless, the routine clinical use of AM is still very limited and is based on single case reports (Mangano et al.).

Combining stem cells with 3D scaffolds may represent a step forward in the pursuit of a faster and improved bone healing. Cells can be directly seeded onto 3D scaffolds before implantation or can be incorporated within biodegradable scaffolds during the manufacturing process using bioprinting techniques (Latimer et al.). As an alternative cell delivery method, cell-encapsulated hydrogel-scaffold constructs have been developed for the controlled release of stem cells at the surgical site. When 3D cell cultures are preferred over monolayer cultures, the encapsulation of the self-assembled cell aggregates within a hydrogel has the advantage to protect the 3D assemblage from disaggregation. In an ectopic bone formation model in mice, a 3D printed polymeric scaffold was found to promote ectopic mineralization to a higher extent when combined with a hydrogel containing spheroid bone marrow-derived stem cells (BMSCs), than when combined with a hydrogel laden with dissociated BMSCs (Shanbhag et al.; Shanbhag et al.).

Other strategies to foster bone tissue ingrowth and mineralization within porous additive manufactured scaffolds rely in the chemical and topographical modification of their surfaces. An acellular organic-inorganic mineralizing construct has been successfully produced, combining a 3D printed nylon scaffold with an elastin-like recombinamer coating, that can be pre-mineralized in the lab prior to implantation (Hasan et al.).

Scaffold bio-functionalization with bioactive agents offers the great advantage of inducing stem-cell homing, while avoiding the several concerns related to stem cell implantation. Among various molecules, the incorporation of bone morphogenetic protein 2 (BMP-2) in 3D scaffolds has been widely explored, demonstrating its ability to trigger stem cell osteogenic differentiation (Zhu et al.). As such, 3D collagen-based matrices functionalized with BMP-2 or with an enamel matrix derivative were found to stimulate *in vitro* the osteogenic commitment of osteoprogenitor cell lines (Lin et al.).

Beside exogenous growth factors, autologous platelet concentrates (APCs) can be employed to enhance the biological properties of bone substitutes. Promising *in vitro* results have been reported in this Research Topic, where the addition of injectable platelet-reach fibrin to different 3D scaffolds positively affected osteoblast cell viability and metabolic activity (Kyyak et al.).

In dentistry, bone augmentation procedures are performed in the vast majority of the cases to restore adequate alveolar ridge dimensions for dental implant placement (Donos et al., 2008; Retzepi and Donos, 2010; Donos et al., 2019). Beside the treatment of alveolar bone deficiencies aiming at replacing missing teeth with implant-supported restorations, the regeneration of the periodontal tissues around compromised teeth as well as of the pulp-dentin complex or of the whole tooth is attracting increasing attention (Latimer et al.). Up-to-date periodontal regeneration strategies include the use of stem cells and the design of multi-material and micropatterned scaffolds, favouring compartmentalized tissue healing and periodontal fiber orientation. Despite the significant advancements noticed in this field, further studies are required to improve the complex multi-tissue periodontal regeneration (Latimer et al.). Furthermore, different tissue engineering strategies, including and not limited to the use of bio-printing, have been introduced to regenerate dental tissues (Cao et al.; Latimer et al.). However, the development of fully formed functional teeth seems far from being achievable in the near future. A deeper understanding of cell-cell and cell-matrix interactions during tooth formation, together with progresses in AM, is needed so that bio-engineered teeth become a clinical reality.

This issue focused mainly in bone regeneration in the oral and maxilla-facial area. However, a broader view on recent developments in the area of orthopaedics was also maintained. Therefore, clinically challenging topics such as non-union fractures and delayed bone healing can also be addressed in the information provided in this issue (Zhu et al.).

Taking into consideration the ageing population and the fact that chronic diseases associated with bone healing and metabolism (i.e., osteoporosis) have high prevalence, novel ways addressing bone healing should be introduced. In this context, the discovery of selective drugs for targeting osteoporosis is particularly relevant. In this issue, an *in vitro* study utilizing BMSCs, leonurine, a natural herbal compound, promoted BMSC osteoblastic differentiation by activating autophagy, making it a potential candidate in the treatment of osteoporosis (Zhao et al.).

However, bone fractures are not limited only to osteoporotic conditions. Special attention has to be devoted to the treatment of pathologies occurring at the growth plate in the paediatric age. This cartilaginous region, which acts as the primary centre for endochondral bone formation in immature long bones, is particularly susceptible to fractures. Current treatments often lead to the development of an undesired bone bridge and to related growth disturbance risks. AM, in combination with cell seeding and active substance delivery, could offer alternative options to existing clinical solutions, in order to achieve cartilaginous tissue reconstruction at the growth plate, thus avoiding the formation of calcified physeal scars (Wang et al.).

Overall, AM, in combination with tissue engineering strategies, offers emerging opportunities in bone regeneration, by enabling the production and the biofunctionalization of customized site-specific 3D scaffold with tunable properties. Looking at the future, remarkable efforts should be directed to the optimization of current technologies and to overcome the hurdles that are delaying the translation of addictive manufactured tissue-engineered bone scaffolds into everyday clinical use.
